# Percent body fat misclassifies patients with sarcopenia as obese: support for fat mass index in obesity assessment

**DOI:** 10.1210/jendso/bvag139

**Published:** 2026-06-24

**Authors:** Susan L Ziolkowski, David R Weber, Joshua F Baker

**Affiliations:** Division of Nephrology, Department of Medicine, Stanford University School of Medicine, Palo Alto, CA 94304, USA; Division of Endocrinology and Diabetes, Department of Pediatrics, Children’s Hospital of Philadelphia, Philadelphia, PA 19104, USA; Division of Rheumatology, Department of Medicine, Perelman School of Medicine at the University of Pennsylvania, Philadelphia, PA 19104, USA; Division of Rheumatology, Department of Medicine, Perelman School of Medicine at the University of Pennsylvania, Philadelphia, PA 19104, USA; Department of Medicine, Corporal Michael J. Crescenz VA Medical Center, Philadelphia, PA 19104, USA; Department of Epidemiology and Biostatistics, University of Pennsylvania, Philadelphia, PA 19104, USA

**Keywords:** body mass index, fat mass index, percent body fat, sarcopenia, obesity

## Abstract

**Background:**

Percent body fat (%BF) is a common measurement reported on dual-energy X-ray absorptiometry (DXA) to assess for obesity. %BF is calculated as fat mass divided by total body mass, and thereby increases with either higher fat mass or reduced muscle mass. In contrast, fat mass index (FMI; kg/m^2^) quantifies fat mass independent of muscle mass. It is unknown whether patients classified as obese by %BF have low muscle mass, and whether individuals with sarcopenia are labeled as obese by %BF vs FMI.

**Methods:**

We utilized whole-body DXA data from 1999-2006 NHANES in adults ≥20 years of age (*N* = 14 850) to estimate the prevalence obesity by %BF, FMI, BMI, and waist circumference. We compared body composition across definitions and examined obesity prevalence among those with sarcopenia.

**Results:**

More patients met criteria for obesity when defined by %BF (76.8%) compared to BMI (33.9%), FMI (35.8%), and WC (51%). Participants classified as obese by %BF had lower lean (muscle) mass, FMI, and BMI compared to participants obese by FMI (all *P* < .001). Among patients with sarcopenia, 62.4% were classified as obese by %BF compared with 0.5% if BMI was used to define obesity.

**Conclusion:**

Patients who are classified as obese by %BF had the lowest muscle mass and fat mass compared to participants who met other definitions of obesity. The majority of patients with sarcopenia are labeled as obese by %BF. We believe these findings support the use of FMI, a measurement not confounded by muscle mass, for research and clinical assessment.

## Short Report

Recent consensus statements now define obesity to include individuals who do not meet traditional body mass index (BMI, kg/m^2^) criteria but are classified as obese based on anthropometric or direct measures of adiposity [1]. Patients identified as obese by anthropometric criteria alone, without a high BMI, have increased risk of organ dysfunction, underscoring the importance of assessing body composition rather than relying on weight-based metrics alone [2].

Dual-energy X-ray absorptiometry (DXA)–derived percent body fat (%BF) is used to assess for obesity. Because %BF is calculated as fat mass divided by total body mass, it cannot distinguish whether high %BF reflects excess adiposity or reduced lean (muscle) mass. DXA-derived fat mass index (FMI; kg/m^2^) [3] overcomes this limitation by isolating fat mass from total body mass; therefore, FMI-based obesity cutoffs are not influenced by muscle mass.

Because %BF may misclassify patients with low muscle as obese, we described body composition in patients meeting criteria for obesity using %BF, FMI, BMI, WC definitions and compared the prevalences of obesity within each group. We also assessed the test characteristics of BMI and waist circumference (WC) to detect high FMI or %BF.

We utilized whole-body DXA data from the 1999-2006 National Health and Nutrition Examination Survey (NHANES) of adults ≥20 years of age (*N* = 14 850). The original data generated and analyzed during this study are included in the Center for Diseases Control and Prevention data repository [4]. Obesity was separately defined as FMI ≥9 kg/m^2^ for men and ≥12 kg/m^2^ for women [3], %BF ≥25 for men and ≥35 for women [5], BMI ≥30 kg/m^2^, or WC ≥102 cm for men and ≥88 cm for women [6]. Muscle mass was assessed by appendicular lean mass index (ALMI, kg/m^2^) derived from DXA and classified as sarcopenic vs nonsarcopenic [7]. Additional information on methods in Supplement 1 [8].

Table 1 summarizes the patient characteristics and prevalences of obesity defined by BMI, WC, %BF, and FMI. Participants classified as obese by %BF had substantially lower ALMI, FMI, and BMI compared to participants obese by FMI (all *P* < .001). More patients met criteria for obesity when defined by %BF (76.8%) compared to BMI (33.9%), FMI (35.8%), and WC (51%).

**Table 1:** Participant characteristics by obesity definition

	Obese by BMI	Obese by waist circumference	Obese by %BF	Obese by FMI
N	5026 (33.9%)	7628 (51.4%)	11 388 (76.8%)	5321 (35.8%)
Age	47.3 (0.34)	49.5 (0.30)	48.2 (0.27)	48.8 (0.31)
Female	54.1%	59.2%	52.4%	51.6%
BMI, kg/m^2^	35.6 (0.11)	32.5 (0.12)	30.3 (0.10)	35.1 (0.12)
FMI, kg/m^2^	14.6 (0.083)	13.1 (0.071)	11.6 (0.064)	14.6 (0.076)
Men	11.9 (0.062)	11.0 (0.052)	9.5 (0.039)	11.8 (0.071)
Women	16.9 (0.055)	14.5 (0.061)	13.5 (0.056)	17.0 (0.054)
ALMI, kg/m^2^	8.9 (0.031)	8.2 (0.035)	7.9 (0.026)	8.7 (0.034)
Men	9.9 (0.028)	9.28 (0.026)	8.7 (0.019)	9.4 (0.030)
Women	8.1 (0.025)	7.39 (0.020)	7.1 (0.019)	8.0 (0.027)
% Body fat	40.6 (0.14)	39.6 (0.10)	37.4 (0.096)	40.9 (0.12)
Men	33.9 (0.096)	33.2 (0.077)	31.3 (0.058)	34.7 (0.070)
Women	46.1 (0.085)	44.0 (0.068)	43.2 (0.072)	44.1 (0.064)
Waist Circ (cm)	112.6 (0.17)	107.8 (0.14)	102.2 (0.13)	112.3 (0.17)
Men	116.0 (0.25)	113.7 (0.19)	105.5 (0.18)	115.0 (0.23)
Women	110.0 (0.23)	103.9 (0.18)	99.1 (0.19)	109.9 (0.23)

Data are presented as mean (SE).

Abbreviations: ALMI, appendicular lean mass index; BMI, body mass index; FMI, fat mass index

**Figure 1:**
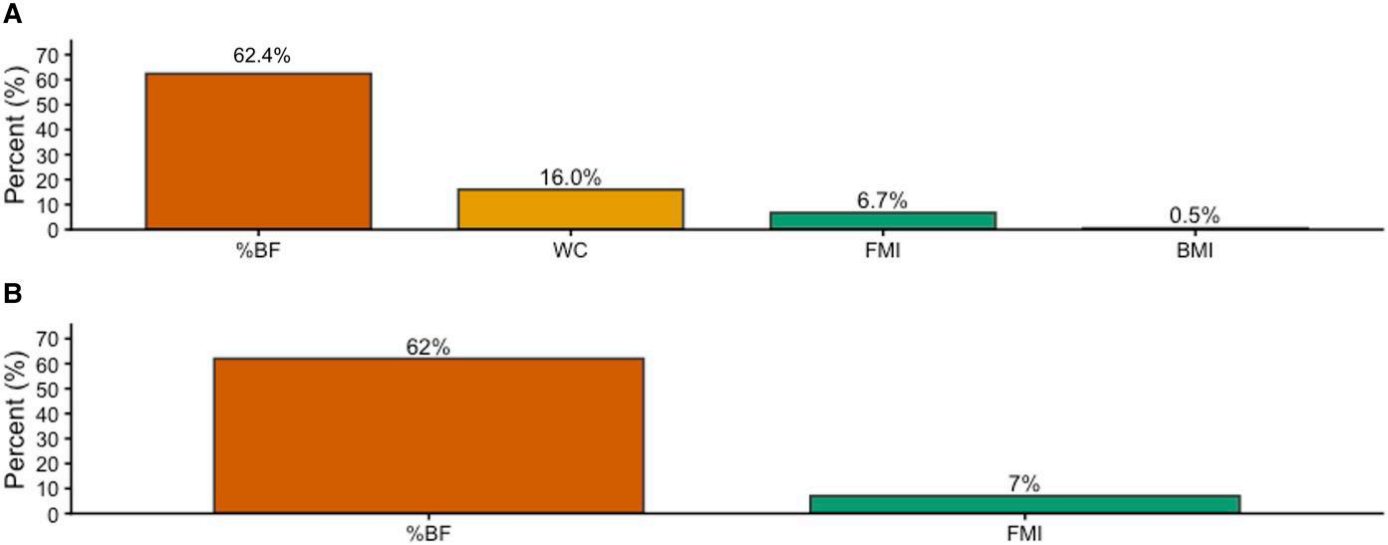
Obesity classification across definitions: (A) proportion of patients with sarcopenia classified as obese by each definition; (B) proportion of BMI-defined nonobese patients meeting obesity criteria by percent body fat or fat mass index.

Figure 1A shows whether patients with sarcopenia were classified as obese using the various definitions of obesity. The majority of patients with sarcopenia, [62.4% (1265/2026)] were classified as obese if %BF was used compared with 0.5% of patients with sarcopenia being classified as obese if BMI was used to define obesity. Comparatively, 6.7% of patients with sarcopenia were classified as obese when using FMI. Among patients defined as obese by %BF, patients with sarcopenia had significantly lower FMI as compared to nonsarcopenic patients [mean (95%CI) sarcopenic FMI = 8.44 (4.80-18.49) vs nonsarcopenic FMI = 11.61 (4.80-41.91, *P* < .001)].

Figure 1B displays whether patients who are classified as nonobese by BMI meet criteria for obesity when defined by %BF or FMI. Among participants nonobese by BMI: 62% were classified as obese by %BF, compared with only 7% by FMI.

Table S1 [8] shows the test characteristics of BMI and WC to detect obesity by %BF or FMI. BMI had low sensitivity to detect obesity when obesity was defined by %BF (43.4%) and NPV was only 37.5% [8]. BMI had higher sensitivity to detect obesity defined by FMI (86.2%) and more balanced PPV and NPV (89.9% and 93%, respectively). WC had slightly lower specificity to detect obesity defined by FMI but similar sensitivity (64.3% and 97.6%, respectively).

Our findings have important implications for both researchers and clinicians and inform how body composition measures should be interpreted in clinical and research settings. Although DXA is not routinely used to screen for obesity in clinical practice, its use has expanded into sports nutrition, athletic training, and research and DXA adiposity measures have been proposed as 1 option to diagnose obesity in recent expert guidance [1, 9]. Many academic and commercial providers offer DXA scans, with %BF commonly reported as the primary body composition metric [10-12]. A prior systematic review reported an association between %BF and mortality [13]; however, the individual contribution of sarcopenia vs excess adiposity to this risk is unclear. Therefore, studies using FMI and ALMI as separate exposures can disentangle these features and clarify their associations with mortality.

It is also important to highlight the favorable test characteristics of BMI to detect obesity as defined by FMI, supporting its role as an effective screening tool. Compared to BMI, WC was more sensitive and less specific. Given the known association between high WC and organ dysfunction in patients with normal BMI [2], clinical screening of WC may also be a beneficial tool because it may identify individuals with excess central adiposity who do not have elevated whole-body FMI.

In summary, the majority of patients with sarcopenia are classified as obese by %BF. Patients classified as obese by %BF had the lowest muscle mass and fat mass compared to other definitions of obesity suggesting an overestimate of adiposity and disproportionate classification of sarcopenic patients as obese. We believe these findings support the use of FMI, a measurement not confounded by muscle mass, rather than %BF in studies on obesity and for confirmatory clinical testing when needed. Given new weight loss therapies, the use of %BF as a confirmation of obesity may be dangerous for many who will also have concurrent muscle loss.

## Data Availability

Original data generated and analyzed during this study are included in this published article or in the data repositories listed in References.
